# The potential for attractive toxic sugar baits to complement core malaria interventions strategies: the need for more evidence

**DOI:** 10.1186/s12936-024-05161-0

**Published:** 2024-11-23

**Authors:** Kennedy Zembere

**Affiliations:** Malawi Liverpool Wellcome Programme, Blantyre, Malawi

**Keywords:** *Anopheles*, Mosquito control, Attractive toxic sugar bait, Residual mosquito control

## Abstract

Despite its success, the increased use of insecticide-treated nets (ITNs) and indoor residual spraying (IRS) has contributed to the development of insecticide resistance in malaria vectors and shifts in biting patterns of the primary malaria vectors. The limitations portrayed by ITNs and IRS suggest that their use alone will not reduce malaria to elimination levels as the remaining untargeted vectors continue to sustain residual malaria transmission (RMT). RMT is a big challenge to malaria elimination because even at 100% ITN and IRS coverage, malaria transmission persists as outdoor vectors avoid or reduce contact with such interventions. With the recent increase in the outdoor biting *Anopheles arabiensis* (hard to control using routine tools), in most African countries, including Malawi, novel tools such as the attractive toxic sugar baits (ATSBs), targeting outdoor biting vectors in addition to controlling indoor vectors are greatly needed to complement current tools, and could facilitate sustainable malaria control. The ATSB is one potential tool that has been tested in different settings with promising results, and more trials are ongoing in other African countries. ATSBs have been attributed to reductions of mosquito densities and malaria incidence with over 80% and 50%, respectively, and there is hope that by 2025, ATSBs would be considered for the World Health Organization prequalification listing as a complementary tool for mosquito control. This article highlights evidence that ATSBs can advance malaria elimination by complementing indoor-based tools. However, for effective control programmes and elimination campaigns, the use of ATSBs alone might not be adequate, and this article recommends the combined use of ATSBs with either IRS or ITNs.

## Background

The wide use of insecticide-treated nets (ITNs) and indoor residual spraying (IRS) for malaria control has significantly contributed to the global reduction of malaria transmission over the past two decades [1, 2]. However, the increased use of pyrethroids in ITNs has resulted in the development of pyrethroid resistance in malaria vectors, leading to reduced efficacy of both ITNs and IRS [3, 4]. Additionally, ITNs and IRS use has contributed to changes in the biting and resting behaviours of *Anopheles* mosquitoes in different areas worldwide, with the endophagic and endophilic behaviours shifting to exophagic and exophilic behaviours, respectively [5, 6] and biting people the early evening before they are protected by nets, sustaining malaria transmission [7, 8]. These severely impact malaria control, contributing to residual malaria transmission (RMT). The impacts of RMT, including sustaining malaria transmission, have been reported in many studies and have been demonstrated even in locations with good ITNs and IRS coverage [9], reducing the possibility that IRS and ITNs alone can achieve malaria elimination [10], calling for novel outdoor control tools that will complement the current tools.

One potential tool that has been tested in different settings with promising results is the attractive toxic sugar bait (ATSB). Contrary to ITNs and IRS, which only target indoor mosquitoes, ATSBs can target both indoor and outdoor mosquitoes [11]. Besides females, ATSBs also target males, exploiting their need for glucose meals [12]. ATSBs comprise an attractive fruit or flower scent (bait), an oral toxin and sugar to attract mosquitoes [13]. This article highlights the potential that ATSBs can advance malaria elimination by complementing the current indoor-oriented tools. The article further highlights how additional evidence could facilitate prequalification of the ATSBs as vector control tools.

## The success and limitations of ITNs and IRS: where is the gap?

ITNs and IRS can significantly reduce malaria transmission by over 90%, reducing malaria incidence and mortality rates [14]. The success of ITNs and IRS has been reported both in Africa and outside of Africa [8, 15]. ITNs and IRS contributed to the reduction of malaria deaths by over 50% globally between 2000 and 2015 [8] and over 663 million malaria cases were prevented in the same period [8]. During the global malaria elimination campaign, which contributed to the elimination of malaria in many parts of the world, IRS and the use of dichloro-diphenyl-trichlorethane (DDT), were the main tools for control according to Pluess et al. [16], and J. Haworth (as cited in [17]).

Despite the success of ITNs and IRS, malaria remains one of the leading causes of mortality, causing over 608,000 deaths in 2022 [18]. This could, in part be due to an increase in malaria transmissions that are sustained by outdoor feeding, early feeding and insecticide-resistant vectors [19]. This has been attributed to increased insecticide resistance and behavioural shifts in many primary vectors because of pyrethroids use, compromising consistency in malaria prevalence reduction [20]. Beier et al. [21] reported that even low and undetectable entomological inoculation rates (EIR) could be responsible for over 20% of malaria prevalence rates. For malaria elimination to be possible, it requires reduction of transmission to levels that are not self-sustaining [22]. The current control tools alone are not able to reduce EIR below 1 per person per year [14, 21]. This evidence suggests that although the current vector control tools are effective, their use alone might not result in malaria elimination as the remaining untargeted vectors continue to sustain transmission.

### Residual malaria transmission (RMT)

RMT is the fraction of total transmission that persists after a full coverage with effective ITNs and/or IRS interventions [23]. RMT is a big challenge to malaria elimination because ITNs and IRS target predominantly indoor mosquitoes; hence even at 100% coverage, malaria transmission persists as outdoor vectors avoid or reduce their contact with ITNs and IRS [19]. Since 2000, the percentage of malaria vectors which bite outdoors had risen by approximately 10% by 2019, and it was predicted that this could increase annual malaria cases by over 10 million [24]. This has been attributed to behavioural shifts of vectors in response to wide use of insecticides [24]. Long time use of insecticides has been associated with increased feeding plasticity in malaria vectors and changes in mosquito feeding behaviours from indoor to outdoor [5, 6, 25, 26]. Increase in outdoor feeding poses a great threat to malaria control as there is no better tool to control outdoor vectors currently, thus, RMT will continue to sustain malaria incidence [27].

## New complementary tools can fill the gap: attractive toxic sugar baits (ATSB)

Mosquitoes primarily depend on plant sugars for their daily survival [28, 29]. Although females also need protein from blood for egg maturation, males solely depend on sugars for their entire life [30]. This has been exploited by developing the ATSB, which contains plant fruit scents to mimic plant sugars and is incorporated with toxins that kill mosquitoes once they feed [13]. Typical ATSBs contain boric acid due to its toxicity to mosquitoes and is regarded safe to human and other mammals [14]. ATSBs have been proposed and experimented in different parts of the world and have since been proven as potential tools that may complement IRS and ITNs in the fight against malaria vectors as they are effective against outdoor residual malaria vectors [14, 31, 32]. Additionally, ATSBs are capable of killing mosquitoes before they complete the extrinsic incubation period, reducing parasite transmission [33, 34]. Although these studies have shown the efficacy of ATSBs, most were small-scale; hence, large-scale studies such as randomized control trials (RCTs) may help to give more evidence.

A cluster randomized control study assessed the impacts of ATSBs in fourteen villages in Mali (2 arms with 7 villages each). They started by collecting baseline mosquito data in all villages, and no significant difference in mosquito abundance was observed between the two arms [35]. After this, an intervention with ATSBs was introduced in one arm. Following the intervention, a significantly lower number of mosquitoes was found in the intervention arm compared to the control arm. The results showed that there was a 57% reduction in mosquito numbers on average (*R* = 0.572, 95% CI 33–72%, *p* < 0.001) [35]. Likewise, ATSB intervention also resulted in a reduction of both indoor and outdoor entomological inoculation rate (EIR) by 89% (95% CI 75–100%, *p* < 0.001) for indoor human landing catch (HLC) and 93% (95% CI 75–100%, *p* < 0.001) for outdoor HLC [35], suggesting that ATSBs are potentially effective tools for both indoor and outdoor vectors. However, the study only presented the EIR data for the wet season with dry season data excluded because it was very small. This could potentially impact the results thus future studies incorporating both seasons may be helpful. Nevertheless, the higher reduction in EIR and reduced sporozoite rates reported in the study indicate that ATSBs have a greater effect on reducing malaria infection rates and possibly, reducing malaria incidence and prevalence. This RCT complements small studies conducted in Asia, America, and Africa [34, 36, 37].

Findings from the first RCT on ATSB in Africa [35] complement what previous small-scale studies found, suggesting that the newly developed ATSB could potentially be a promising tool in addition to the current vector control tools. Since ATSBs are effective against indoor and outdoor mosquitoes, this may significantly impact malaria control and possibly contribute to elimination. Additionally, insecticides in ATSBs are directly taken through feeding, increasing the insecticides’ fast action, unlike ITNs or IRS, which require contact [35]. As such, this may reduce the possibilities of insecticide resistance, thus valuable for insecticide resistance management for integrated vector management programmes. Although the potential for insecticide resistance in ATSBs is low, the likelihood of resistance particularly for long-term use cannot be taken out and hence, using ATSBs with two or more different toxins may help reduce resistance emergence [14]. The World Health Organization (WHO) also recommends that when using both IRS and ITNs together for maximum control, the insecticides used in ITNs and IRS must be from different classes to minimize the potential for resistance [38] with a non-pyrethroid insecticide (e.g. carbamates) used in IRS more especially in areas with pyrethroid resistant vectors [39]. This knowledge could also be useful when implementing ATSBs for vector-borne disease control.

To date, there is only one published RCT [35] that has reported a statistically significant impact of ATSBs on malaria vectors in Africa, from the search of the literature. There is a need for more RCTs on ATSBs in different countries of Africa to complement the available data. With the most recent RCT (preprint) reporting a non-significant impact of ATSBs on reducing *Anopheles funestus* density in Zambia [40], additional large scale RCTs in different contexts and regions are still critical. Currently, there are large scale trials that are ongoing in Mali and Kenya [41]. The trial settings in Mali and Kenya differ from those in Zambia in different ways, including the malaria vector species composition and distribution of natural sugar sources, factors that may influence ATSB efficacy [40]. There is great hope that data generated from the current RCTs could complement on the already available data, thus, facilitating the prequalification of the ATSBs by the WHO. It is expected that by 2025, the ATSB would possibly achieve the WHO-prequalification listing as a complementary malaria control tool [42], thus more evidence on its effectiveness in different geographical settings would help to facilitate this process.

To achieve the global vector borne disease control targets, the need for deploying a full potential of vector control tools is required [7, 8]. The capacity that ATSBs have in incorporating novel insecticides other than pyrethroids could increase their efficacy. This could increase their impact, killing pyrethroid-resistant mosquitoes in the presence of ITNs, making them an additional potential candidate for managing insecticide resistance [31]. The ATSB has great potential to be used as part of integrated vector management programmes to supplement current tools and this article recommends combined use of ATSBs with ITNs and IRS. The effects of combined use of ATSBs and ITNs have been shown previously where up to 100% reduction in EIR was observed when ATSBs were used together with ITNs [35].

## ATSBs could facilitate the malaria elimination goals in Malawi

Over the years, entomological studies in Malawi have shown that *Anopheles arabiensis* is the major malaria vector in the country [35–37, 40, 41]. Due to its crepuscular and plastic feeding behaviours, *An. arabiensis* is hard to control, when ITNs and IRS are the only control tools being used [24]. This has resulted in year-round malaria transmission, even with the successful implementation of traditional control tools (IRS and ITNs) [43]. Novel mosquito control tools targeting outdoor and indoor biting vectors are greatly needed to facilitate sustainable malaria control in Malawi. Since Malawi, Zambia and Kenya and most of the eastern African countries share the same species of mosquitoes [27, 44–46], there could be hope that Malawi could benefit from the ATSB study results from Kenya and the neighbouring Zambia to guide future rollout and implementations of the ATSBs in the country. However, due to differences in geographical factors and other factors, there is still a need for ATSB trials in a Malawian local context to guide proper implementations of the same for practical use.

## Conclusion and recommendations

ATSBs have a great potential to facilitate malaria elimination efforts by complementing the current control tool kit. This article recommends additional evidence-based research on ATSBs in different countries of Africa to complement the available data. This would increase the likelihood that ATSBs could be incorporated to complement ITNs and IRS as part of integrated vector management.

## Data Availability

Not applicable.

## Abbreviations

ASTB: Attractive toxic sugar baits
ITNs: Insecticide treated nets
IRS: Indoor residual spraying
RMT: Residual malaria transmission
RCT: Randomized control trials
EIR: Entomological inoculation rate
HLC: Human landing catch
CI: Confidence interval
WHO: World Health Organization
